# A Systematic Way to Infer the Regulation Relations of miRNAs on Target Genes and Critical miRNAs in Cancers

**DOI:** 10.3389/fgene.2020.00278

**Published:** 2020-03-31

**Authors:** Peng Xu, Qian Wu, Jian Yu, Yongsheng Rao, Zheng Kou, Gang Fang, Xiaolong Shi, Wenbin Liu, Henry Han

**Affiliations:** ^1^Institute of Computational Science and Technology, Guangzhou University, Guangzhou, China; ^2^School of Computer Science of Information Technology, Qiannan Normal University for Nationalities, Duyun, China; ^3^Department of Computer and Information Science, Fordham University, New York, NY, United States

**Keywords:** miRNA, influence, cancer, target gene, regulation relation

## Abstract

MicroRNAs (miRNAs) are a class of important non-coding RNAs, which play important roles in tumorigenesis and development by targeting oncogenes or tumor suppressor genes. One miRNA can regulate multiple genes, and one gene can be regulated by multiple miRNAs. To promote the clinical application of miRNAs, two fundamental questions should be answered: what's the regulatory mechanism of a miRNA to a gene, and which miRNAs are important for a specific type of cancer. In this study, we propose a miRNA influence capturing (miRNAInf) to decipher regulation relations of miRNAs on target genes and identify critical miRNAs in cancers in a systematic approach. With the pair-wise miRNA/gene expression profiles data, we consider the assigning problem of a miRNA on target genes and determine the regulatory mechanisms by computing the Pearson correlation coefficient between the expression changes of a miRNA and that of its target gene. Furthermore, we compute the relative local influence strength of a miRNA on its target gene. Finally, integrate the local influence strength and target gene's importance to determine the critical miRNAs involved in specific cancer. Results on breast, liver and prostate cancers show that positive regulations are as common as negative regulations. The top-ranked miRNAs show great potential as therapeutic targets driving cancer to a normal state, and they are demonstrated to be closely related to cancers based on biological functional analysis, drug sensitivity/resistance analysis and survival analysis. This study will be helpful for the discovery of critical miRNAs and development of miRNAs-based clinical therapeutics.

## Introduction

MicroRNAs (miRNAs) are a class of small non-coding RNAs and have been proved to play important roles in regulating more than two thirds of human genes (Bandyopadhyay et al., 2010; Song et al., 2017). They usually regulate their target genes by binding to the complementary seed sequence at the 3′ untranslated region. The binding of miRNAs usually leads to the translation repression or degradation of the target mRNAs and ultimately affects the production of the corresponding proteins (Bartel, 2009; Fabian et al., 2010; Hata and Lieberman, 2015). For example, miR-21 was demonstrated to negatively regulate the expression of SAV1 (Sun et al., 2019) and Smad6 (Xu et al., 2016) in colorectal cancer. One single miRNA usually targets many genes and one gene might be regulated by multiple miRNAs. To decipher the relationships between miRNAs and their target genes and unveil miRNAs' biological functions, many miRNA targets databases, such as Targetscan (Agarwal et al., 2015), miRDB (Wong and Wang, 2015), miRanda (Betel et al., 2010), and mirTarbase (Chou et al., 2018), have been built based on various biological experiments and/or different computation methods.

The dysfunctions of miRNAs have been reported to be involved in the tumorigenesis of various cancers (Bartel, 2004; Gotte, 2010; Dela Cruz and Matushansky, 2011; Lovat et al., 2011; Liu W. et al., 2018; Xu et al., 2018). For this reason, miRNAs have become potential biomarkers in cancer diagnosis and treatment (Slack and Chinnaiyan, 2019). Furthermore, some miRNA-based therapeutics have entered into the clinical research, i.e., miR-16-based mimics in phase I clinical trial for treating advanced non-small cell lung cancer, and antimiRs targeted at miR-122 in phase II trial for treating hepatitis (Rupaimoole and Slack, 2017).

However, accumulating evidence indicates that miRNAs can also promote the expression of their target genes. For example, Vasudevan et al. found that miR396-3 could direct the AGO complex binding with the AU-rich elements to promote the translation of its target gene in Vasudevan et al. (2007). They further demonstrated that let-7 and synthetic miRcxcr4 could induce target mRNAs up-regulation on cell cycle arrest while repressing translation in proliferation cells (Vasudevan and Steitz, 2007). In addition to functioning in the cytoplasm, mature miRNAs are also found in the nucleus. Xiao et al. demonstrated that miR-24-1 in the nucleus can activate gene transcription by targeting their enhancers (Xiao et al., 2017). Up to now, more than 200 positive regulations of miRNAs on genes have been experimentally identified in the literature.

It becomes a fundamental problem to elucidate the regulatory relations between miRNAs and their target genes in systems biology. Specifically, we need to know which genes are positively regulated by one miRNA and which genes are negatively regulated by it. The answer to this problem will provide a foundation to study the critical roles of miRNAs in tumorigenesis. Recently, Tan et al. *first* investigated this problem based on the Pearson correlation coefficients between the expression of miRNAs and their target genes in pan-cancer datasets (Tan et al., 2019). Surprisingly, they found many positive correlated miRNA-gene pairs. This demonstrates that miRNAs could exert their important roles in various cancers by positively regulating many genes.

Another important issue is to determine the critical miRNAs potential to affect the overexpression or under-expression of cancer-related genes. The answer to this question will help to determine a few “level point” miRNAs for designing miRNA-based therapeutic strategies. Cui et al. combined the miRNA sequence features and miRNA disease spectrum width (DSW) to define the importance of miRNAs (Cui et al., 2019). However, this static definition could not reflect the different regulatory mechanisms between miRNAs and genes involved in specific cancer well.

In this paper, we propose a novel miRNA influence capturing (MiRNAInf) to decipher regulation relations of miRNAs on target genes and identify critical miRNAs in cancers in a systematic approach. We study miRNA-gene regulations by assuming that the expression of one gene is determined by its upstream miRNAs. We model the expression of a gene as a function of the expression of the miRNAs targeting it. Through the Taylor expansion, we employ the first partial derivative to a miRNA to denote its regulatory effect on the target gene. The first partial derivative is then approximated by the Pearson correlation coefficient of the expression change of a miRNA and that of its target gene between disease and normal control.

We finally define the global influence of a miRNA by combining its local influence strength in an individual cancer and the degree of its target gene in a PPI network. Our results on breast cancer, prostate adenocarcinoma and liver cancer datasets further demonstrate that positive regulations are as common as negative ones in miRNA-gene interactions. We also find that only a few miRNAs have significant influences on the cancer-related differentially expressed genes. The identified top miRNAs in the three datasets are not only highly correlated in a functional network, but also significantly enriched in some important functions such as inflammation, cell proliferation, apoptosis and cell cycle. It demonstrates that they are very likely to play essential roles coherently in tumorigenesis. More importantly, we find that the intervention of a few critical miRNAs may alleviate the abnormal expressions of most genes according to the regulatory effect and differential expression situation between miRNAs and their target genes. Besides, the identified important miRNAs influence patients' survival time of prognosis besides the sensitivity/resistance of some anti-cancer drugs. In sum, our study provides a systematic way to understand the key roles of miRNAs in cancers and to screen potential intervention miRNA biomarkers for future miRNA-based therapy and diagnosis in precision medicine.

## Materials and Methods

### Data Acquisition and Preprocessing

We collected three types of data: miRNA and gene expression data for three cancers, miRNA-gene interaction data, and protein-protein interaction (PPI) data from three different databases: TCGA, miRTarbase and STRING (Chou et al., 2016; Szklarczyk et al., 2017).

#### The Cancer Genome Atlas (TCGA) Data

We collected datasets with both miRNA and gene expression profiles for cancer samples and corresponding normal samples in this study. Three cancers with abundant gene expression and miRNA expression “pair datasets” were acquired from TCGA (http://tcga-data.nci.nih.gov/tcga/). They included 102 samples for breast cancer, 52 samples for prostate adenocarcinoma, and 49 samples for liver cancer.

#### MiRNA-Gene Interaction Data

Besides gene expression and miRNA expression data, we further downloaded miRNA-gene targets data from miRTarbase (Chou et al., 2016), a widely-used state-of-the-art database for miRNA-gene targets. miRTarbase includes 502,652 high quality experimentally validated miRNA-gene interactions between 2,599 miRNAs and 15,064 genes for the human species.

#### Protein-Protein Interaction (PPI) Data

The STRING database, which includes 10,048,286 interactions between the 19,576 proteins for human beings, integrates the experimentally validated and computationally predicted protein-protein interactions (Szklarczyk et al., 2017). In order to use the highly confident interactions, we selected the interactions with a combined score >150. The distribution of proteins' degrees indicates that a small number of proteins have interactions with hundreds of other nodes, while most proteins only have interactions with a few of other proteins, which satisfies power-law distribution (Please go to Figure S1 for details).

### Methods

**Figure 1:**
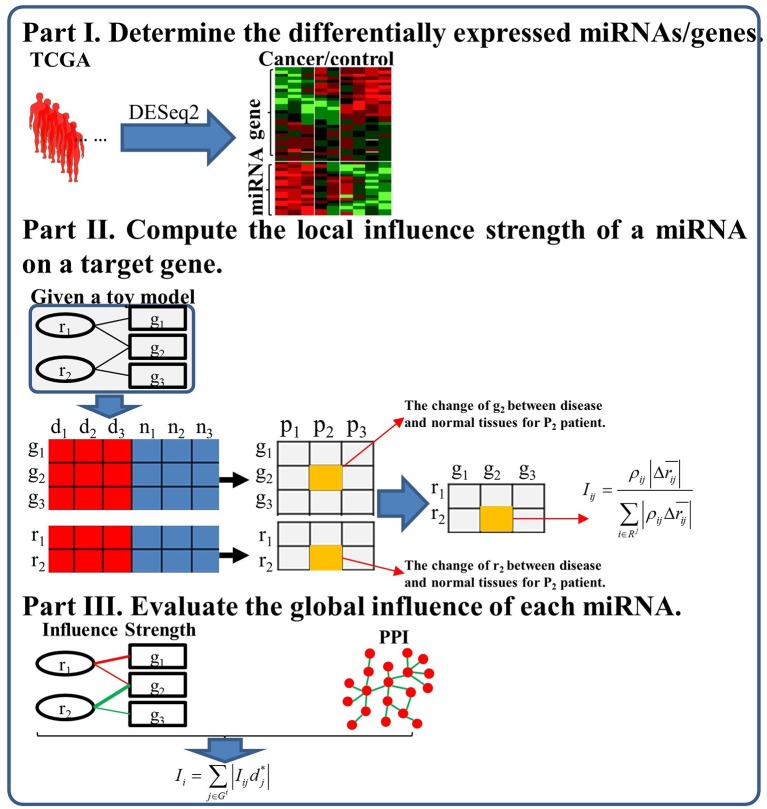
The flowchart of the proposed study framework.

In this study, we propose a miRNAInf method to identify critical miRNAs involved in cancer as well as conducting comprehensive functional analysis for miRNAs. The flowchart of the proposed miRNAInf methods is illustrated in Figure 1. The proposed method consists of the three steps. First, we determine the significant differentially expressed miRNAs and genes based on their expression data from TCGA. Second, we compute the local influence strength of a miRNA to its target gene. Finally, we evaluate the global influence of a miRNA in a specific cancer by integrating the local influence strength and gene's importance.

#### Identify Differentially Expressed miRNAs and Genes

We conduct normalization for miRNA and gene expression data before identifying differentially expressed miRNAs and genes. For each miRNA epression sample, we apply the RPM (Reads Per Million) method for normalization as described in Equation (1) for its simplicity and efficiency (Faraldi et al., 2019).

(1) $$\begin{array}{r} r_{i}^{RPM}=\frac{E_{r_{i}}}{Total_{r}}\times 10^{6} \end{array}$$

where *E*_*r*_*i*__ means the read counts of *miRNA* *i* and *Total*_*r*_ indicates the total counts of all miRNAs in a specific sample. RPM normalizes all read counts with respect to the ratio between library size and a million number. Similarly, we normalize gene expression data as follows

(2) $$\begin{array}{r} g_{j}^{RPM}=\frac{E_{g_{j}}}{Total_{g}}\times 10^{6} \end{array}$$

where *E*_*g*_*j*__ means the read counts of *gene* *j* and *Total*_*g*_ indicates the total counts of all genes in a specific sample.

We then apply DESeq2 (Love et al., 2014) to identify differentially expressed genes (DEGs) and differentially expressed miRNAs (DEmiRs) by collecting all genes and miRNAs with *p* < 0.05 adjusted by FDR method in differential expression analysis of the normalized data. Finally, we get 546,457 and 313 DemiRs and 5,057, 3,665, 3,064 DEGs for breast, liver, and prostate cancer, respectively.

#### Compute miRNA Local Influence Strength on a Target Gene

The expression of a gene can be assumed as a function of the expression of the miRNAs targeting it. Given a gene *j* regulated by *m* miRNAs *r*_1_, *r*_2_, …*r*_*i*_, …*r*_*m*_, then its expression $g_{j}=f^{j}\left(r_{1},r_{2},\ldots r_{i},\ldots r_{m}\right)$ in disease state can be approximated by first-level Taylor expansion as

(3) $$\begin{array}{r} f^{j}\left(r_{1},r_{2},\ldots r_{i},\ldots r_{m}\right)=f^{j}\left(r_{0}^{1},r_{0}^{2},\ldots r_{0}^{i},\ldots r_{0}^{m}\right)\\ \;+\underset{i\in R^{j}}{\sum }\frac{\partial f^{j}\left(r_{1},r_{2},\ldots r_{i},\ldots r_{m}\right)}{\partial r_{i}}\Delta r_{i} \end{array}$$

where $f^{j}\left(r_{0}^{1},r_{0}^{2},\ldots r_{0}^{i},\ldots r_{0}^{m}\right)$ is the expression value of gene *j* in normal state, *R*^*j*^ is the index set of the miRNAs regulating gene *j*, Δ*r*_*i*_ = $r_{i}-r_{0}^{i}\left(i\in \left\{1,2,\ldots ,m\right\}\right)$ is the change of miRNA *i* between the disease state and normal state.

The gene expression change Δ*g*_*j*_ of gene *j* can be calculated by moving the gene expression in the normal state $f^{j}\left(r_{0}^{1},r_{0}^{2},\ldots r_{0}^{i},\ldots r_{0}^{m}\right)$ to the left side of Equation 3:

(4) $$\begin{array}{r} \Delta g_{j}=f^{j}\left(r_{1},r_{2},\ldots r_{i},\ldots r_{m}\right)-f^{j}\left(r_{0}^{1},r_{0}^{2},\ldots r_{0}^{i},\ldots r_{0}^{m}\right)\\ \;=\underset{i\in R^{j}}{\sum }\frac{\partial f^{j}\left(r_{1},r_{2},\ldots r_{i},\ldots r_{m}\right)}{\partial r_{i}}\Delta r_{i} \end{array}$$

where the right side represents the sum of expression change of gene *j* induced by the perturbation of each miRNA *i*. The partial derivative $\frac{\partial f^{j}\left(r_{1},r_{2},\ldots r_{i},\ldots r_{m}\right)}{\partial r_{i}}$ actually reflects the influence strength of miRNA *i* on gene *j*. A given miRNA *i* may target many other genes, we assume that Δ*r*_*ij*_, the portion of the *i*th miRNA expression difference Δ*r*_*i*_ between the disease and normal states, affecting gene *j*, is positively proportional to the expression change Δ*g*_*j*_. Given the target gene index set *G*^*i*^ of miRNA *i*, we define Δ*r*_*ij*_ as the product of the absolute change ratio of gene *j* $\left|\Delta g_{i}|/\underset{j\in G^{i}}{\sum }|\Delta g_{j}\right|$ and the change of miRNA *i* between the disease state and normal state Δ*r*_*i*_:

(5) $$\begin{array}{r} \Delta r_{ij}=\frac{\left|\Delta g_{j}\right|}{\underset{j\in G^{i}}{\sum }|\Delta g_{j}|}\times \Delta r_{i} \end{array}$$

Based on this, we can calculate the Pearson correlation coefficient ρ_*ij*_ between Δ*r*_*ij*_ and Δ*g*_*j*_. ρ_*ij*_ > 0 indicates that miRNA *i* upregulate gene *j*, otherwise, it suppresses gene *j*. Then the partial derivative $\frac{\partial f^{j}\left(r_{1},r_{2},\ldots r_{i},\ldots r_{m}\right)}{\partial r_{i}}$ can be approximated by

(6) $$\begin{array}{r} \frac{\partial f^{j}\left(r_{1},r_{2},\ldots r_{i},\ldots r_{m}\right)}{\partial r_{i}}=k_{ij}\rho_{ij} \end{array}$$

where the coefficient *k*_*ij*_ can be assumed and approximated by a constant *k* for all miRNA-genes.

Given one miRNA *i*, it may have different influence strengths on each target gene. Thus, we compute its local influence strength *I*_*ij*_ as follows

(7) $$\begin{array}{r} I_{ij}=\frac{\rho_{ij}|\bar{\Delta r_{ij}}|}{\underset{i\in R^{j}}{\sum }|\rho_{ij}\bar{\Delta r_{ij}}|} \end{array}$$

where the $\bar{\Delta r_{ij}}$ means the average difference between disease and normal tissues for all patients. The local influence strength *I*_*ij*_ considers both the correlation coefficient ρ_*ij*_ and the average difference $\bar{\Delta r_{ij}}$. The higher correlation ρ_*ij*_ between them, the larger its influence on gene *j*. Similarly, the larger average difference $\bar{\Delta r_{ij}}$, the larger also its influence on gene *j*.

#### Evaluate the Global Influence of Each miRNA

In order to describe the importance of a miRNA in a specific disease, we consider both the number of its target genes and the importance of each gene. Here, we define the global influence of a miRNA *i* for the disease by weighting its local influence by the importance of its target genes:

(8) $$\begin{array}{r} \begin{array}{c} I_{i}=\underset{j\in G^{i}}{\sum }|I_{ij}d_{j}^{*}|\\ \;=\underset{j\in G^{i}}{\sum }|I_{ij}\frac{d_{j}}{d_{\max}}| \end{array} \end{array}$$

where *d*_*j*_ and *d*_max_ represent the degree of the gene *j* and the maximum degree in the PPI network, respectively. The importance of the gene *j* is modeled as the ratio of its degree between the maximum degree in the PPI: $\frac{d_{i}}{d_{\max}}$. The global influence of a miRNA *i* involves both the local influence strength and the importance of its target genes. The more targets regulated by miRNA *i*, the larger its global influence. Simultaneously, the larger the degrees of its target genes in PPI, the larger the global influence.

## Results

### Positive Regulations Are as Common as Negative Ones in Cancers

**Figure 2:**
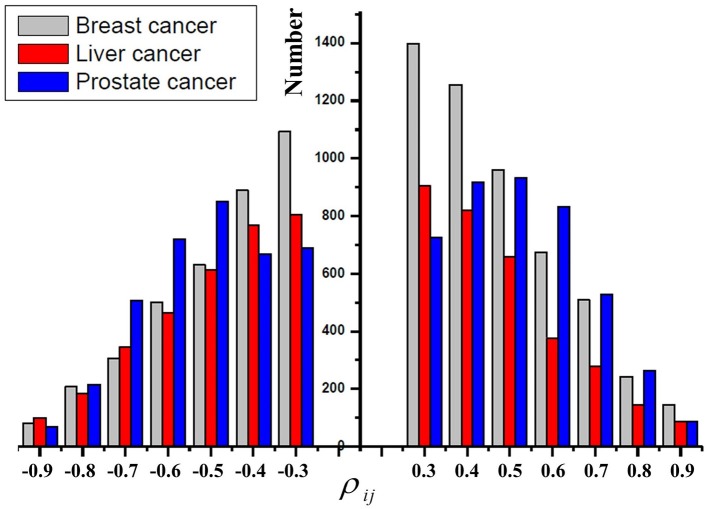
The distribution of the Pearson correlation coefficients ρ_*ij*_ in breast cancer, liver cancer, and prostate cancer, respectively.

As mentioned before, it suggests that positive regulations also play important roles in cancers. We first study the distribution of the Pearson correlation coefficient ρ_*ij*_ between the change of a miRNA and that of its target genes. Figure 2 shows the distribution of the number of |ρ_*ij*_|> 0.3 in breast cancer, liver cancer, and prostate cancer, respectively. First, the distribution of the number of ρ_*ij*_> 0.3 and that of ρ_*ij*_ < −0.3 are very similar. Our observation further supports the results in Tan et al. (2019) that miRNAs exert both positive and negative regulations on their target genes. Second, most of the absolute ρ_*ij*_ are smaller than or equal to 0.5, which indicates that most of the regulatory strengths are relatively weak because one miRNA may target even hundreds of genes; On the other hand, only a few absolute |ρ_*ij*_| are larger than 0.8, which indicates that several individual genes may be strongly regulated by very few miRNAs.

**Figure 3:**
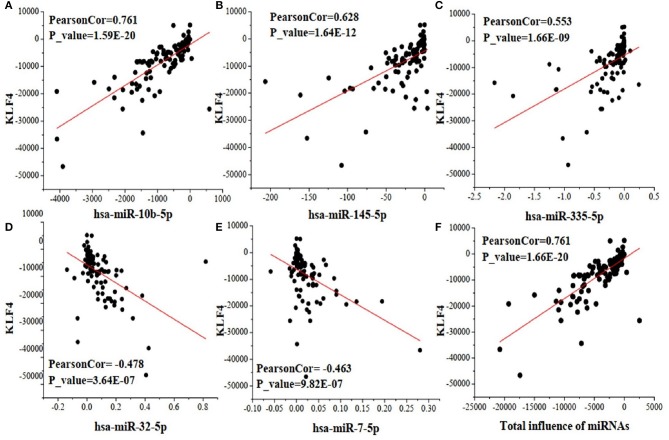
The scatter plots between the change of five miRNAs and that of their target gene KLF4. **(A)** the correlation between hsa-miR-10b-5p and KLF4; **(B)** the correlation between hsa-miR-145-5p and KLF4; **(C)** the correlation between hsa-miR-335-5p and KLF4; **(D)** the correlation between hsa-miR-32-5p and KLF4; **(E)** the correlation between hsa-miR-7-5p and KLF4; **(F)** the correlation of total influence of the five miRNAs and their target gene KLF4.

As an example, Figure 3 shows the scatter plots between the change of five miRNAs and that of their target gene KLF4, which regulates many critical physiologic and cellular processes (Wang et al., 2018). The X-axis of Figures 3A–E represents the expression change of a specific miRNA binding on target gene KLF4, X-axis of Figure 3F represents the total change of five miRNAs regulating KLF4, and the Y-axis of Figure 3 represents the expression change of KLF4. We can see that three miRNAs (hsa-miR-10b-5p, hsa-145-5p, and hsa-miR-335-5p) positively correlate with KLF4, while the other two miRNAs (hsa-32-5p and hsa-miR-7-5p) negatively correlate with it. This demonstrates that miRNAs targeting one gene may affect it differently. Their total expression changes positively correlate with that of KLF4 as shown in the last subplot of Figure 3. It indicates that some impacts of the negatively correlated miRNAs can be offset by those of dominant ones.

### miRNAs Regulate Their Target Genes in a Complex Way

**Figure 4:**
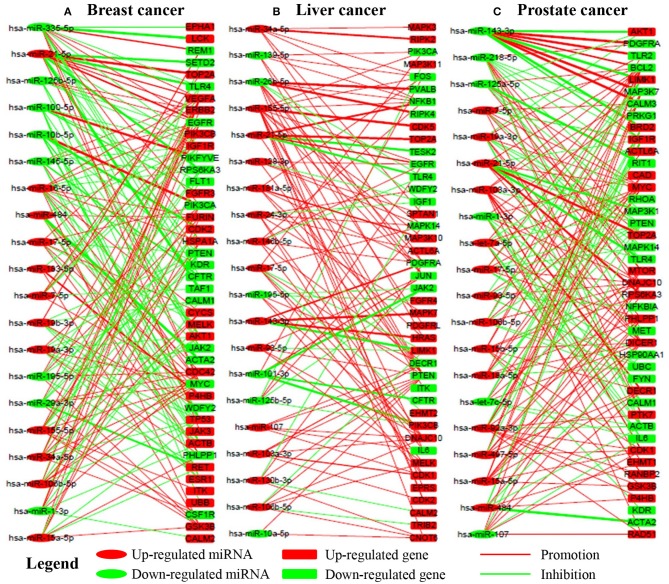
The regualtory networks between miRNAs and genes in breast, liver, and prostate cancer, respectively. Red and green lines indicate upregualtion and downregualtion, respectively. Line width indicates the absoulte value of the local influence strength. Red and green nodes indicate overexpression and underexpression, respectively. **(A–C)** are the subnetworks for breast, liver and prostate cancer respectively.

In this section, we select a portion of miRNAs and their target genes and display their local influence relation in a bipartite graph in Figure 4. We can see that one miRNA may promote the expression of some genes while repressing that of the others. On the other hand, one gene may be upregulated by some miRNAs while being downregulated by other miRNAs. Furthermore, one miRNA may have a larger influence (wide lines) on some genes while having relatively smaller influence (thin lines) on the others. The observations demonstrate that miRNAs interact with their target genes in a complex way. The inference of these complex interactions forms the basis for us to understand the detailed roles of each miRNA on a specific gene. For example, PIK3CA is regulated by hsa-miR-10b-5p (Influence strength, 0.9509), hsa-miR-335-5p (Influence strength, 2.83E-4), hsa-miR-17-5p (Influence strength,-7.75E-4), hsa-miR-19a-3p (Influence strength,-7.47E-5), and hsa-miR-155-5p (Influence strength,-7.29E-4). Then its expression is thus mainly upregulated by hsa-miR-10b-5p.

### Only a Few miRNAs Have Significant Global Influences on Cancers

From the perspective of systems biology, we are more interested in the most critical miRNAs, i.e., dominant miRNAs that have the greatest influence on the whole regulatory network. Identifying the dominant miRNAs will answer the key question: which miRNAs are regulators of the most cancer-related genes?

**Figure 5:**
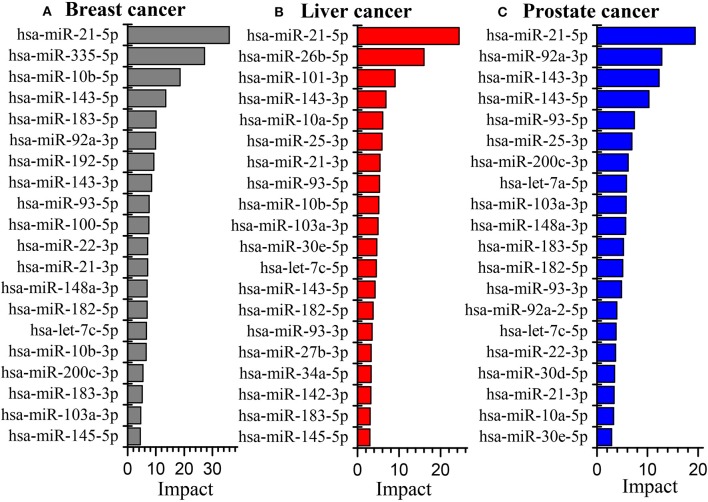
The global influence of the top 20 miRNAs in breast cancer **(A)**, liver cancer **(B)** and prostate cancer **(C)** datasets, respectively.

Figure 5 illustrates the global influence of the top-ranked 20 miRNAs in breast, liver and prostate cancers, respectively. We can see that some miRNAs appear in all the three cancers whereas others may only show in one or two cancers. It suggests that some miRNAs play a common important role in many cancers while others are more related to specific cancers. Moreover, there are only a few miRNAs whose global influences are extremely larger than those of the others. This indicates their dysfunction may have very crucial impacts on the development of cancers. For instance, miR-21 ranked as the top one in the three cancers, has been confirmed highly involved in cancer proliferation and metastasis (Liu H. et al., 2018; Wang et al., 2019). On the other hand, we find that the most influenced genes by the dysfunctional miRNAs are highly related with cancers, such as CDK2, TP53, HRAS, NFKB1 (Carroll et al., 2000; Normanno et al., 2009; Xu et al., 2016).

## Functional Analysis of the Critical miRNAs

### Intervention of a Few Critical miRNAs May Help to Alleviate the Abnormal Expression of Most Cancer-Related Genes

Because one miRNA may regulate multiple downstream genes and the intervention on it may have different effects on the expression of its target genes. From the perspective of miRNA-based therapy, it is very crucial to figure out how the intervention of one miRNA may affect the abnormal expression of their target genes. If a miRNA promotes the expression of a gene and they are both overexpressed or under-expressed, then the intervention of the miRNA will exert a positive effect on the target gene to alleviate its abnormal expression; If a miRNA represses the expression of a gene and they have an opposite abnormal expression situation, then the intervention of the miRNA will also exert a positive effect on the target gene to alleviate the abnormal expression. Conversely, if a miRNA promotes the expression of a gene and they have the opposite abnormal expression situation, then the intervention of the miRNA will exert a negative effect on the target gene to deteriorate its abnormal expression. On the other hand, if a miRNA represses the expression of a gene and they are both overexpressed/under-expressed, then the intervention of the miRNA will also exert a negative effect on the target gene to deteriorate its abnormal expression.

**Figure 6:**
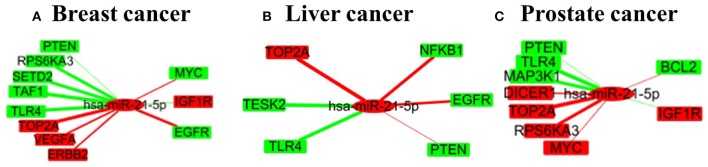
A small subnetwork of miRNA-21-5p and its target genes in three cancers. **(A–C)** are for the subnetworks in breast cancer, liver cancer and prostate cancer respectively.

Figure 6 shows the subnetwork of hsa-miR-21-5p in the three cancers, when intervention on hsa-miR-21-5p, the left-hand genes are those being positively affected while the right-hand ones negatively affected genes. This reveals that the intervention of one miRNA may have complex effects on cancer-related genes. Specifically, the abnormal expression of some genes can be alleviated while the other may be further deteriorated.

Based on the regulation relations and the abnormal expression situations, we summarize the number of positively and the negatively affected miRNA-gene pairs of the top five miRNAs in Table 1. The “+”/“-” symbols in Table 1 represent the number of positively/negatively affected miRNA-gene pairs after intervention. We have the following observations from the table. First, we can see that the interventions of the five miRNAs may affect about 1,000 of abnormal miRNA-gene pairs which indicate they regulate many downstream genes. Second, the number of positively affected genes is extremely larger than that of the negatively affected ones. Third, the absolute relative influence strengths of most of the positively affected pairs are larger than 0.5. The observations indicate that the intervention of the top five miRNAs may significantly drive the cancer-related genes to the normal levels. Therefore, they are potential invention biomarkers for miRNA-based therapy.

**Table 1:** The number of positive and negative effect interactions between the top 5 miRNAs and their targets in three cancers, respectively.

***Abs*(*I*_*ij*_)**	**Breast cancer**		**Liver cancer**		**Prostate cancer**	
	**+**	**–**	**+**	**–**	**+**	**–**
**(0.9–1)**	**583**	**42**	**175**	**35**	**344**	**2**
**(0.8–0.9)**	**190**	**9**	**92**	**16**	**119**	**1**
**(0.7–0.8)**	**94**	**4**	**76**	**25**	**86**	**1**
**(0.6–0.7)**	**90**	**13**	**63**	**18**	**58**	**0**
**(0.5–0.6)**	**84**	**19**	**48**	**15**	**64**	**3**
**(0.4–0.5)**	77	15	66	28	67	2
**(0.3–0.4)**	44	19	54	37	71	5
**(0.2–0.3)**	43	30	52	50	82	4
**(0.1–0.2)**	52	50	61	59	158	17
**Total**	1257	201	687	283	1049	35

*The bold values are the number of interactions with influence strength more than 0.5*.

### Most of the Critical miRNAs Involve in Some Important Biological Functions

We conduct functional analysis for top-ranked miRNAs by integrating the co-expression similarity, co-GO similarity, co-literature similarity, and co-similar disease similarity by using miRNA functional analysis tool MISIM (Li et al., 2019). There are 14, 15, and 13 miRNAs respectively annotated by MISIM in the top 20 important miRNAs of the three cancer datasets.

**Figure 7:**
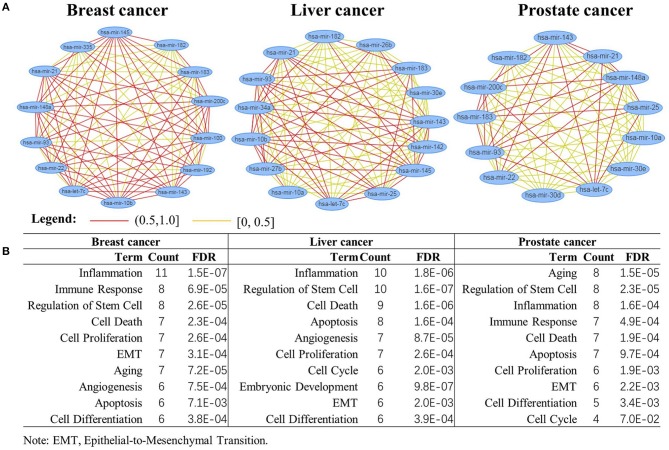
Biological function analysis of the top 20 miRNAs. **(A)** miRNAs function enrichment analysis; **(B)** functional similarity network analysis of the miRNAs.

Figure 7A shows the function similarity network of the top 20 miRNAs, where red color lines denote the correlation coefficients larger than 0.5. It suggests most of the miRNAs are highly correlated in their biological functions. Figure 7B shows the top 10 enriched biological functions (FDR<7.0E-02) of the miRNAs. These biological functions, such as inflammation, cell proliferation, apoptosis, and cell cycle, have been verified closely related to different cancers (Evan and Vousden, 2001; Taniguchi and Karin, 2018; Xu et al., 2020). This indicates the critical miRNAs might interact in a highly coherent way to drive the biological system from normal to disease state in the three cancers.

As the overlapped critical miRNA in three cancers, hsa-mir-21 was reported to involved in various cancers such as colorectal cancer, breast cancer and lung cancer (Xu et al., 2016; Liu W. et al., 2018). It has been reported that overexpression of miR-21 could promote the cellular proliferation, colony formation, invasion and also inhibit cell death in a wide variety of cancerous cells by regulation of various targets including PTEN, TPM1, and PDCD4 (Najjary et al., 2020).

### Some Critical miRNAs Also Impact the Resistance/Sensitivity of Drugs

Some non-coding RNAs (ncRNAs) especially miRNAs could promote sensitivity or produce resistance of drugs by regulating their target genes. To evaluate the impacts of the critical miRNAs on the resistance/sensitivity of drugs, we submit the top 20 miRNAs to two state-of-the-art miRNA-drug interaction databases: *ncDR* (Dai et al., 2017) and *mTD* (Chen et al., 2017). The two databases include 1,056, 384, and 127 records for miRNA-drug interactions in breast, liver and prostate cancers respectively as well as curating a lot of resistance/sensitivity related ncRNAs.

**Figure 8:**
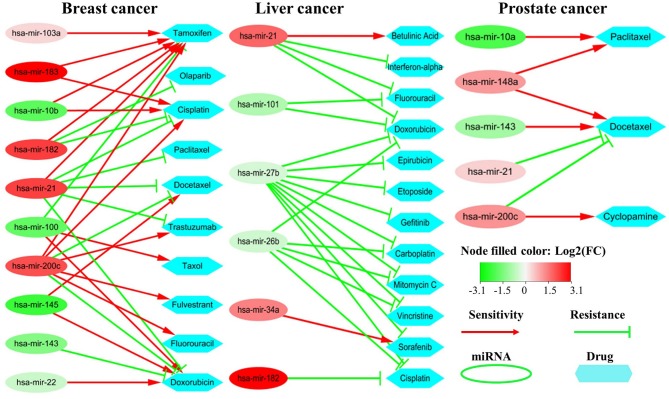
Drug sensitivity/resistance analysis of the top 20 miRNAs. The red (or green) oval represents the miRNA is up-regulated (or down-regulated) in cancer samples. The red line denotes promoting drug sensitivity while the green line denotes inducing drug resistance.

We find that there are 10, 6, and 4 miRNAs impacting drug resistance/sensitivity cases in breast cancer, liver and prostate cancer, respectively, among the top 20 miRNAs. The main reason for the small number of miRNAs in the liver and prostate cancers lies in that there are relatively fewer records about them in the two databases. Figure 8 shows their abnormal expression and corresponding influence on drug sensitivity/resistance. On one hand, one miRNA may influence multiple drugs with different effects. For example, the over-expression of hsa-mir-182 in breast cancer could induce drug resistance to both Olaparib and Cisplatin while promoting the sensitivity of Tamoxifen. On the other hand, some miRNAs may promote a drug sensitivity while others induce its resistance. The complex effects of these miRNAs on cancer-related drugs not only further demonstrate their importance in cancer development, but also provide a new insight for accurate drug selection.

### The Expression of Critical miRNAs Is Highly Related to the Survival Time of Prognosis

**Figure 9:**
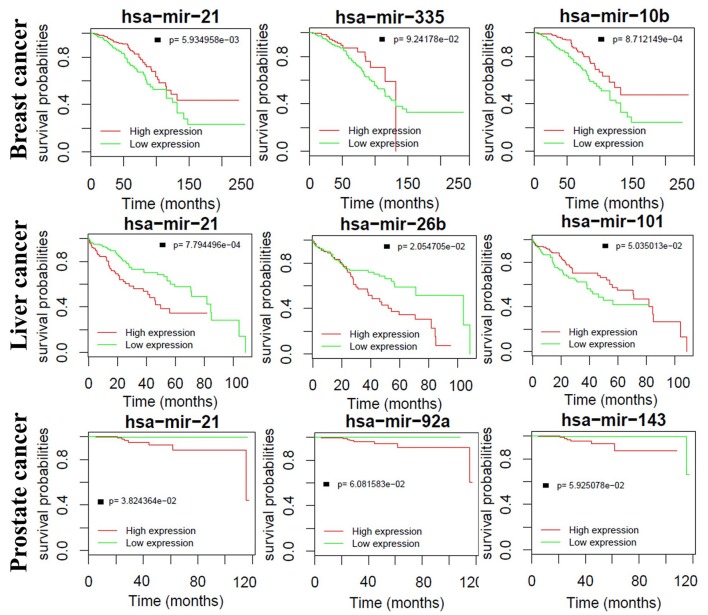
Kaplan–Meier curves of the top three miRNAs in breast, liver and prostate cancers, respectively.

We also find that critical miRNAs can influence the survival time of prognosis seriously. Figure 9 shows the Kaplan–Meier curves of the top three miRNAs in breast, liver and prostate cancers, respectively. Most of them are significantly correlated to the overall survival time in both breast and liver cancers except for prostate cancer. One major reason is that most prostate tumors are slow-growing and many of them are not lethal. Furthermore, some important correlations between the miRNAs are supported by wet-lab experiments. For example, Yan et al. demonstrated that overexpression of miR-21 was associated with human breast cancer poor prognosis (Yan et al., 2008). Ji et al. showed that liver cancer patients with low miR-26 expression had shorter overall survival time (Ji et al., 2009, 2013). These observations indicate that the identified critical miRNAs may also serve as potential biomarkers for the survival time of prognosis.

## Conclusion

MiRNAs have been reported as a kind of important non-coding regulators influencing the expression of more than 60% genes. In this paper, we proposed a novel miRNA influence capturing (miRNAInf) method to characterize the regulatory mechanism of miRNA on their target genes as well as identify critical miRNAs that have dominantly important impacts on target genes. Out results from the breast, prostate and liver cancer datasets further verify that miRNAs may either upregulate or downregulate their target genes instead of mainly repressing them. We identified some critical miRNAs involved in the three cancers by constructing a miRNA-gene regulatory network. Our biological functional analysis shows that those critical miRNAs are not only highly correlated with each other but also involved in many important biological functions such as apoptosis, proliferation, etc. Furthermore, miRNA-gene interaction analysis reveals that the intervention of only a few top crucial miRNAs may potentially alleviate the abnormal expressions of many genes and push the cancer system to a normal situation. It suggests that the identified crucial miRNAs may serve as potential biomarkers for miRNA-based therapy as well as diagnosis. In addition, we find some critical miRNAs may influence the sensitivity/resistance of drugs as well as the survival time of prognosis. Our study provides a strong foundation to support the combination of miRNA-based therapy and cancer drugs to improve the treatment effect in precision medicine. To the best of our knowledge, this study first provides a systematic approach to decipher the roles of miRNAs in the diagnosis and prognosis of complex diseases and will inspire future studies in this field.

## Data Availability Statement

All datasets generated for this study are included in the article/Supplementary Material. The source codes for this study can be found at https://github.com/xupeng2017/MiRNAInf.

## Author Contributions

WL and PX designed the methods. PX wrote the codes. WL, PX, QW, JY, YR, GF, ZK, XS, and HH analyzed the data. WL, HH, and PX wrote the manuscript. All authors read and approved the final manuscript.

### Conflict of Interest

The authors declare that the research was conducted in the absence of any commercial or financial relationships that could be construed as a potential conflict of interest.
