# Supplementary material for: A Systematic Way to Infer the Regulation Relations of miRNAs on Target Genes and Critical miRNAs in Cancers
Source: Front Genet. 2020 Mar 31;11:278. doi: 10.3389/fgene.2020.00278 (PMC7136563; doi:10.3389/fgene.2020.00278)
Supplement: Supplementary file 1 [file Table_1.DOCX]

**Supplementary File**

**A systematic way to infer the regulation relations of miRNAs on target genes and critical miRNAs in cancers**

Peng Xu^1,2^, Qian Wu^1^, Jian Yu^1^, Yongsheng Rao^1^, Zheng Kou^1^, Gang Fang^1^, Xiaolong Shi^1^, Wenbin Liu^1*^, Henry Han^3*^

^1^Institute of computational science and technology, Guangzhou University, Guangzhou, Guangdong, 510006, China.

^2^School of computer science of information technology, Qiannan Normal University for Nationalities, Duyun, Guizhou, 558000, China.

^3^Department of computer and information science, Fordham University, New York, NY, 10023, USA.

*Correspondence author: wbliu6910@126.com, [xhan9@fordham.edu](mailto:xhan9@fordham.edu)


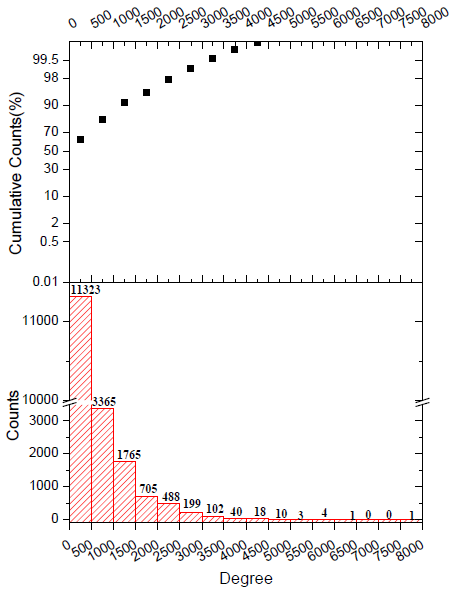


Figure S1. String database：PPI: 19576 nodes, 11353056 interactions . The protein ID in PPI network were converted to Gene symbol ID by R software package “biomaRt”, al last 10048286 interactions were got between the 18014 nodes.
